# Neuronal Activity Distributed in Multiple Cortical Areas during Voluntary Control of the Native Arm or a Brain-Computer Interface

**DOI:** 10.1523/ENEURO.0376-20.2020

**Published:** 2020-10-26

**Authors:** Zheng Liu, Marc H. Schieber

**Affiliations:** 1 Department of Biomedical Engineering, University of Rochester, Rochester, NY 14627; 2 Department of Neurology, University of Rochester, Rochester, NY 14642

**Keywords:** brain-computer interface, effective connectivity, posterior parietal cortex, premotor cortex, primary motor cortex, primary somatosensory cortex

## Abstract

Voluntary control of visually-guided upper extremity movements involves neuronal activity in multiple areas of the cerebral cortex. Studies of brain-computer interfaces (BCIs) that use spike recordings for input, however, have focused largely on activity in the region from which those neurons that directly control the BCI, which we call BCI units, are recorded. We hypothesized that just as voluntary control of the arm and hand involves activity in multiple cortical areas, so does voluntary control of a BCI. In two subjects (*Macaca mulatta*) performing a center-out task both with a hand-held joystick and with a BCI directly controlled by four primary motor cortex (M1) BCI units, we recorded the activity of other, non-BCI units in M1, dorsal premotor cortex (PMd) and ventral premotor cortex (PMv), primary somatosensory cortex (S1), dorsal posterior parietal cortex (dPPC), and the anterior intraparietal area (AIP). In most of these areas, non-BCI units were active in similar percentages and at similar modulation depths during both joystick and BCI trials. Both BCI and non-BCI units showed changes in preferred direction (PD). Additionally, the prevalence of effective connectivity between BCI and non-BCI units was similar during both tasks. The subject with better BCI performance showed increased percentages of modulated non-BCI units with increased modulation depth and increased effective connectivity during BCI as compared with joystick trials; such increases were not found in the subject with poorer BCI performance. During voluntary, closed-loop control, non-BCI units in a given cortical area may function similarly whether the effector is the native upper extremity or a BCI-controlled device.

## Significance Statement

Reaching to and grasping a visible object involves neuronal activity in multiple areas of the cerebral cortex. Whether neurons in these areas are engaged similarly when a subject controls a brain-computer interface (BCI) remains unknown. We found similar unit activity in multiple cortical areas as subjects performed a center-out task with either a hand-held joystick or a BCI controlled by only four BCI units in the primary motor cortex (M1). Like the four BCI units, non-BCI units in most cortical areas showed changes in their preferred direction (PD) between joystick and BCI trials, with similar modulation depths and effective connectivity. We suggest that a given cortical area may function similarly during voluntary closed-loop control of either the upper extremity or a BCI.

## Introduction

Voluntary control of movement involves many parts of the central and peripheral nervous system. In the mammalian cerebral cortex, active regions include the primary motor cortex (M1), dorsal premotor cortex (PMd), ventral premotor cortex (PMv), primary somatosensory cortex (S1), dorsal posterior parietal cortex (dPPC), and anterior intraparietal area (AIP; Rizzolatti et al., 1998; Grafton, 2010). While neural activity in M1 is primarily responsible for movement execution, concurrent activity in these additional frontal and parietal areas may be involved in receiving information on goal/target selection for movement planning, in computing feedforward models of the expected movement, and in processing feedback from the ongoing movement (Shadmehr and Krakauer, 2008).

Brain-computer interfaces (BCIs) now are being developed not only to control prosthetic arms (Hochberg et al., 2012; Wodlinger et al., 2015; Lebedev and Nicolelis, 2017; Andersen et al., 2019), but also to investigate nervous system function (Jarosiewicz et al., 2008; Law et al., 2014; Sadtler et al., 2014; Moxon and Foffani, 2015; Golub et al., 2018; Oby et al., 2019; Zhou et al., 2019). The majority of BCI studies to date that employ neuron recordings have focused on analyzing the activity of those neurons that contribute directly to the decoded BCI output, which we refer to as “BCI units.” These BCI units comprise only a small fraction of the neuronal population active locally, however. Other simultaneously recorded neurons, which we refer to as “non-BCI units,” have been found to be active along with BCI units, sometimes changing their patterns of activity in ways similar to the BCI units (Hwang et al., 2013; Arduin et al., 2014; Law et al., 2014). With few exceptions (Koralek et al., 2012; Bridges et al., 2020), however, these non-BCI units have been in the same cortical area as the BCI units.

Yet just as voluntary control of natural upper extremity movement requires the participation of cortical areas beyond M1, controlling a closed-loop BCI is likely to require the activity of neurons beyond the BCI units. The BCI units at least must receive processed visual information on the location of the goal/target, and probably are affected by processed visual feedback on the motion of the effector as well. Decisions about when to initiate the next trial, when to start the motion of the effector, and when to stop, all must reach the BCI units. The firing of the BCI units may also be processed as efference copy, being compared with an internal model of the expected feedback. Indeed, as human subjects learned to modulate high-γ ECoG potentials at one electrode in the motor or premotor cortex, strong parallel activation in prefrontal, premotor, and posterior parietal cortex appeared and then diminished as learning progressed (Wander et al., 2013). Whether features of unit activity such as preferred direction (PD) and modulation depth change in non-BCI units distributed across multiple cortical areas remains unknown.

We hypothesized that controlling a BCI would entail activity not only of the BCI units but also of non-BCI units distributed throughout the multiple cortical areas that participate in natural control of upper extremity movement. Furthermore, if controlling a BCI required changes in the natural activity patterns of the BCI units, then the activity patterns of non-BCI units, in terms of PD, modulation depth, and effective connectivity, potentially could change as well. We therefore trained monkeys already experienced in a joystick-controlled center-out task to perform a similar task with a novel BCI. Rather than using a BCI decoder optimized to incorporate the natural tuning of large numbers of M1 neurons (Athalye et al., 2017; Zhou et al., 2019), we chose a BCI decoder that used only four M1 neurons, each assigned arbitrarily to drive velocity in the one of the four cardinal directions. We considered it likely that this decoder, while difficult to learn, would require novel patterns of coactivation among the BCI units and out-of-manifold reorganization involving non-BCI units as well (Fetz, 1969; Moritz et al., 2008; Nazarpour et al., 2012; Law et al., 2014; Oby et al., 2019). Moreover, rather than pursuing extensive BCI training to achieve performance equivalent to that with the hand-held joystick, we chose instead to train a more permissive task less extensively but repeatedly with different sets of BCI units, enabling us to distinguish consistent versus inconsistent features in the activity of non-BCI units. After the monkeys acquired a preselected level of proficiency with each set of BCI units, we compared the activity of non-BCI units in M1, PMd, PMv, S1, dPPC, and AIP during joystick control versus BCI control.

## Materials and Methods

### Subjects

Two male rhesus monkeys, Q and P (weighing 9–11 kg), were subjects in the present study. All procedures for the care and use of these nonhuman primates followed the National Institutes of Health Guide for the Care and Use of Laboratory Animals and were approved by the University Committee on Animal Resources at the University of Rochester, Rochester, New York.

### Center-out task

Each monkey initially was trained to perform a two-dimensional center-out task using a joystick held with the right hand to control the position of a cursor. The base of the joystick was inclined 30° toward the primate chair in which the monkey was seated. When centered, the joystick knob was positioned 20 cm in front of and 5 cm below the monkey’s right shoulder. The monkey then could reach all positions within a 20 × 20 cm hand workspace. An LCD screen positioned 90 cm in front of the monkey at eye level displayed both the cursor and the targets in a square visual workspace divided into 1000 “screen units” horizontally and 1000 screen units vertically.

**Figure 1.**
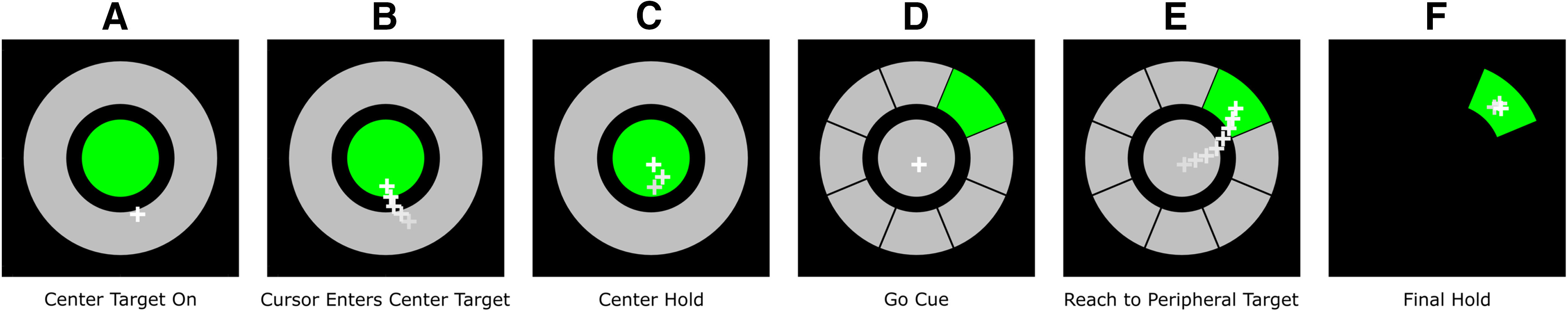
Center-out task. The monkey controlled the movement of the cursor (white “+”) from the center to the peripheral target in the follow task sequence: ***A***, The center target turned green. ***B***, The monkey positioned the cursor in the center target. ***C***, The monkey maintained the cursor in the center target for an initial center hold epoch. ***D***, The center target turned gray as a Go Cue, and simultaneously one of the eight peripheral targets turned green, instructing a cursor movement to that target. ***E***, The monkey moved the cursor into the instructed (green) target. ***F***, Except for the instructed, green target, the display then turned black and the monkey thereafter maintained the cursor in the green target for a final hold epoch, the end of which completed a successful trial.

Trials began when the circular center target turned green (Fig. 1*A*). The monkey then positioned the cursor (white “+”) within the center target (Fig. 1*B*) and kept the cursor there for a 500 ms center hold epoch (Fig. 1*C*). Rather than traditional circular peripheral targets, we used eight segments of an annulus, eliminating the possibility that the cursor could pass between targets. When the center hold epoch ended, the center target turned gray and one of the eight peripheral targets turned green (Fig. 1*D*), providing a Go cue that instructed the monkey to move the cursor out of the center target within 2000 ms and into the instructed peripheral target within another 2000 ms, without entering any other peripheral target (Fig. 1*E*). The center target and the other seven peripheral targets then disappeared, and monkey was required to keep the cursor in the peripheral target for a final hold epoch lasting 600 ms (Fig. 1*F*). The end of the final hold period was designated a Success event. Successful trials were rewarded with a drop of water. If any of these conditions was not met, however, the trial was aborted immediately, the entire display turned red, and a 1500 ms error timeout followed. Every trial was followed by a 1500 ms intertrial interval, after which the center target re-appeared and the subject then could initiate the next trial. Trials were presented in blocks including 1 each of the eight peripheral targets presented in a random order that was re-randomized between blocks. To prevent the monkey from rejecting trials involving particular targets, error trials were repeated until performed successfully. The entire behavioral task was controlled by custom software running on a PC which also sent behavioral event marker codes into the collected data stream.

### Microelectrode arrays

**Figure 2.**
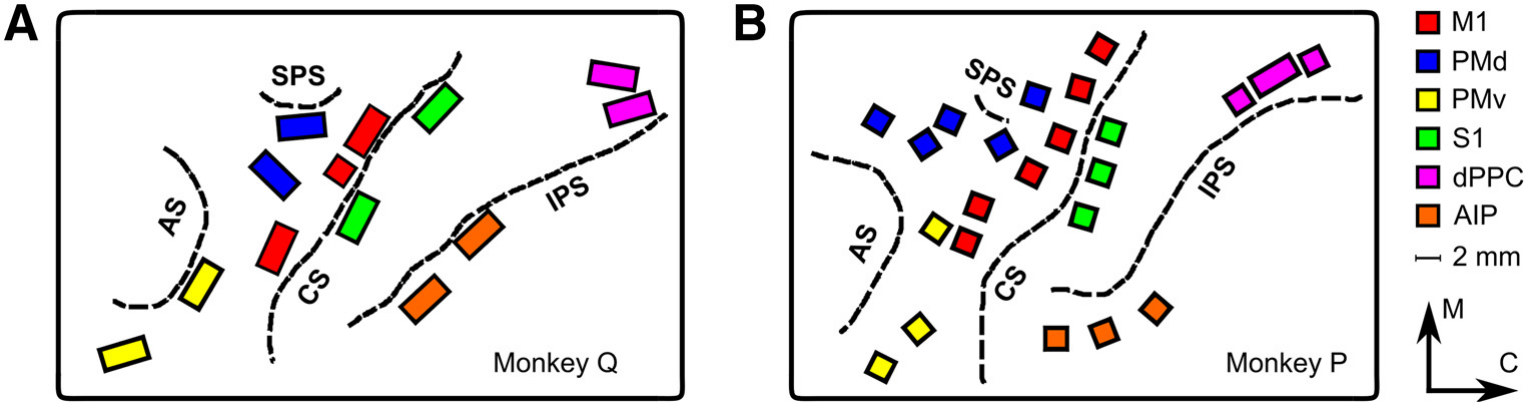
Location of arrays. ***A***, Monkey Q. ***B***, Monkey P. Array locations and cortical sulci were redrawn from intraoperative photographs. CS, central sulcus; AS, arcuate sulcus; SPS, superior precentral sulcus; IPS, intraparietal sulcus. Orientation arrows: M, medial; C, caudal.

Once each monkey was trained to perform the center-out task, floating microelectrode arrays (FMAs; MicroProbes for Life Sciences) were implanted in six cortical areas using procedures described in detail previously (Mollazadeh et al., 2011; Rouse and Schieber, 2016). Figure 2 shows the location of the arrays implanted in M1, PMd, PMv, S1, dPPC, and AIP. Note that our dPPC arrays recorded from the medial intraparietal area (MIP) in the anterior bank of the intraparietal sulcus as well as adjacent parts of area PE on the surface of the postcentral gyrus (Bakola et al., 2017; Rajalingham and Musallam, 2017; De Vitis et al., 2019). Except for AIP, all these areas are known to be active during center-out movements (Georgopoulos et al., 1982; Kalaska et al., 1983; Prud'homme et al., 1994; Batista and Andersen, 2001; Stark et al., 2007; Rajalingham and Musallam, 2017; De Vitis et al., 2019). For Monkey Q, two 32-channel FMAs were implanted in each of the six cortical areas, and an additional 16-channel FMA was implanted in M1. For Monkey P, six 16-channel FMAs were implanted in M1, five in PMd, three in PMv, three in S1, two in dPPC, and three in AIP; and an additional 32-channel array was implanted in dPPC. The length of electrodes varied from 1 to 8 mm. All electrodes were made from 70% Pt and 30% Ir and had a nominal impedance of 0.4–0.6 MΩ at the time of implantation.

### Neural data acquisition

Neural data were collected with two 128-channel Plexon data acquisition systems (Plexon Inc.) and one 128-channel Cerebus data acquisition system (Blackrock Microsystems). For Monkey Q, all implanted electrodes could be recorded simultaneously with the three recording systems. For Monkey P, however, one of the Plexon systems was not available, and the total number of implanted electrodes (384) was greater than the number of recording channels available (256). Premotor and parietal arrays therefore were grouped separately and recorded on alternate days along with the M1 arrays (group 1: M1, PMv, and PMd arrays; group 2: M1, S1, AIP, dPPC arrays).

Signals from each FMA were amplified by a head stage (gain 20× for the Plexon systems, 2× for the Cerebus system) and further amplified by the data acquisition hardware before being digitized and stored to disk by the host PCs for each system. For the Plexon systems, spike waveforms that crossed a threshold selected interactively on-line were sampled at 40 kHz using Sort Client (Plexon), with additional sorting off-line. For the Cerebus system, broadband signals were sampled at 30 kHz, and spikes were extracted off-line and sorted with custom MATLAB (MathWorks) scripts. Both single units and multi-units were included in analyses, whereas any sorted units with a signal-to-noise ratio (SNR) < 2.5 were discarded. Behavioral event marker codes generated by the task control PC were used to synchronize the data recorded by different acquisition systems for analysis.

### BCI control

BCI experiments were performed 6–14 months after surgical implantation of microelectrode arrays. Daily recording sessions started with ∼200 joystick-controlled trials, followed by trials in which the monkey’s arm was restrained in the primate chair and the cursor was controlled through a BCI. Neural data were recorded continuously throughout both joystick-controlled and then BCI-controlled trials.

To control the BCI, four units were chosen randomly from a pool of ∼10–20 candidate M1 units (either single units or multi-units) that had been recorded stably on-line for at least 5 d. These four units were assigned randomly by a MATLAB script to drive cursor velocity in the four cardinal directions: rightward, leftward, upward, or downward. The directional assignment of each of these BCI units was made without consideration of its PD during joystick trials.

The firing rate of each BCI unit controlled the output of a separate linear velocity decoder that moved the cursor in the assigned cardinal direction. The output of each velocity decoder at time t, v(t), was calculated as:

(1) $$v\left(t\right)=\frac{A}{\sqrt{FR^{80\%}}-\sqrt{FR^{20\%}}}\times \left(\sqrt{FR\left(t\right)}-\sqrt{FR^{20\%}}\right)-\frac{A}{2},$$

where FR(t) is the instantaneous firing rate of the BCI unit estimated using spike counts in 10 ms bins convolved with a 500 ms Gaussian filter and A is an empirical value set to 6 screen units per 10 ms. Square-root transformation of the unit’s firing rate was used to reduce variance (Kihlberg et al., 1972; Ashe and Georgopoulos, 1994; Rouse and Schieber, 2016). The 80th and 20th percentiles of the BCI unit’s firing rate distribution, $FR^{80\%}$ and $FR^{20\%}$, were estimated initially from the cumulative distribution of firing rates recorded during joystick-controlled trials before beginning the BCI task each day. To adjust for firing pattern changes between joystick control and BCI control, $FR^{80\%}$ and $FR^{20\%}$ were updated after the first 5 min of BCI control using the cumulative distribution of firing rates during that interval. To prevent surges on individual BCI-unit decoders, v(t) was limited to the range of ±3 screen units/10 ms. The cursor’s horizontal and vertical velocities then were determined independently, each in a “push-pull” fashion based on the output of the four velocity decoders driven separately by the four BCI units ($v_{right}$, $v_{left}$, $v_{up}$, $v_{down}$):

(2) $$v_{horizontal}=v_{right}-v_{left}$$

(3) $$v_{vertical}=v_{up}-v_{down}.$$

For example, if $v_{right}$ = 2.5, $v_{left}$ = −0.3, $v_{up}$ = −0.2, $v_{down}$ = −2.3, then $v_{horizontal}$ = 2.8, $v_{vertical}$ = 2.1, and the resultant cursor velocity was 3.5 ($=\sqrt{2.8^{2}+2.1^{2}}$) screen units/10 ms at 37° [= $tan^{-1}(2.1/2.8$)]. Cursor position was updated every 10 ms.

### BCI training

As expected from previous work (Law et al., 2014; Sadtler et al., 2014; Oby et al., 2019), learning to control the cursor with four arbitrarily assigned M1 units proved challenging for both monkeys. We therefore relaxed the criteria for successful trial completion to a level at which the monkeys persevered in learning to control the BCI rather than becoming excessively frustrated. The center hold epoch was reduced to 20 ms; the final hold epoch was reduced to 50 ms; the subjects were allowed up to 5000 ms to move the cursor out of the center target once the peripheral target had appeared, and 5000 ms more to move the cursor into the correct peripheral target after leaving the center target. In addition, during the movement to the peripheral target (Fig. 1*E*), the subjects were allowed to enter peripheral targets other than the instructed target. Each monkey then could be trained over several days to control the BCI using the four M1 units each acting in their arbitrarily assigned direction. If a BCI unit driving cursor motion in a given direction was lost to isolation during this period, we assigned another M1 unit to that direction using the same criteria described above.

**Figure 3.**
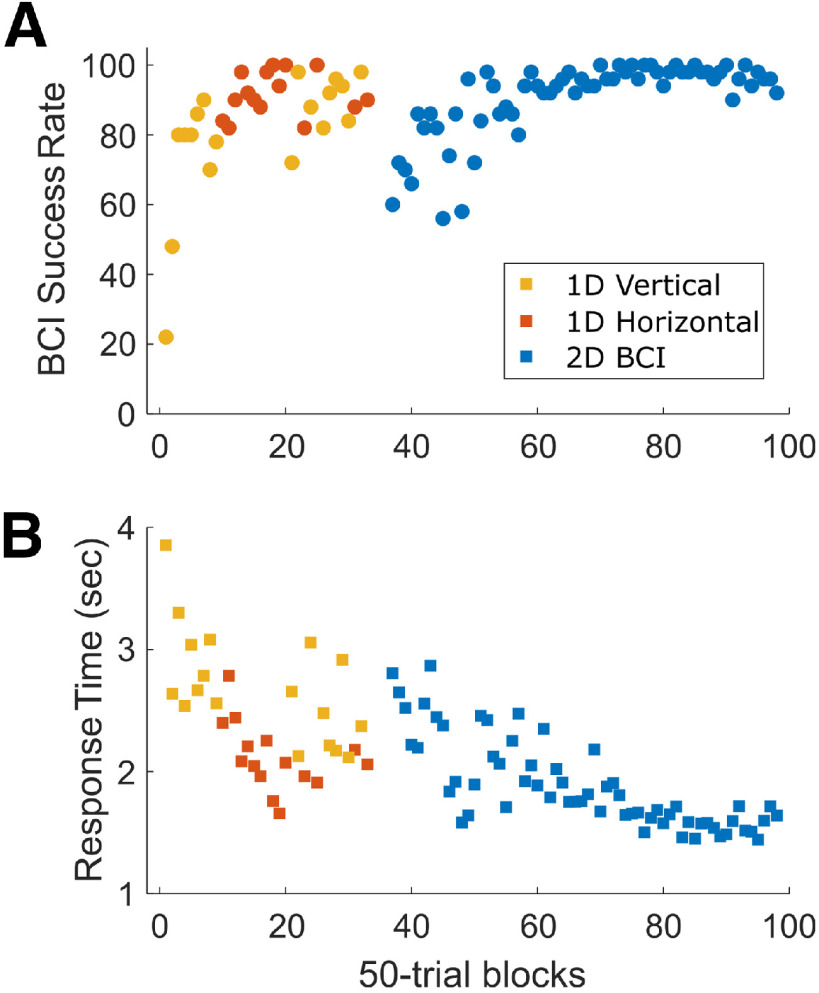
BCI training. Success rates (***A***) and mean response times (***B***) are shown in sequential blocks of 50 successfully performed trials over 10 daily training sessions. As success rates increased, response times (from the appearance of the Go cue until the cursor had been in the target for 50 ms) decreased. The number of blocks of 50 successful trials varied from one training day to the next. Data from Monkey Q, assignment ii.

Figure 3 illustrates the progress of BCI training in sequential blocks of 50 correctly performed trials. Training began with a one-dimensional (1D) BCI task, initially presenting only the upward and downward targets (Fig. 3, 1D vertical) and then only the rightward and leftward targets (Fig. 3, 1D horizontal). One-dimensional training continued for 5 d (block 33) until the monkey performed successfully in ∼80% of the trials for each dimension separately. Thereafter, the monkey was trained to control two-dimensional cursor movement to all eight peripheral targets, reaching a plateau of relatively stable performance with success rates >80% after another few days (block 58). As the success rate rose, the response time, from the Go cue until the end of the final hold epoch, fell.

We refer to the set of four M1 units selected to simultaneously control the BCI as a BCI-unit “assignment.” Once a subject had reached a stable plateau at success rates >80% and data had been collected with one such assignment, a new BCI-unit assignment was selected randomly using the criteria described above, with the additional restriction that a given M1 unit was not assigned to control the same direction in more than one assignment. The BCI training process then was repeated, starting with one-dimensional BCI training and progressing to two-dimensional training, and additional data were collected after the monkey’s performance again had reached a plateau at success rates >80%. Monkey Q was trained to this level with five different BCI-unit assignments, and Monkey P with three. Initial one-dimensional training required an average of 5.6 ± 3.1 d (mean ± SD across BCI-unit assignments) for Monkey Q and 5.7 ± 0.6 d for Monkey P. Two-dimensional training then required another 6.4 ± 1.1 d for Monkey Q and 9.0 ± 3.6 d for Monkey P.

### Experimental design and statistical analyses

For each BCI-unit assignment, we analyzed neural data collected once the subjects were performing the two-dimensional BCI task at a success rate >80%. We selected for analysis sessions in which the subject had performed at least 400 BCI trials successfully. These sessions also included ∼200 successfully performed joystick-controlled trials. To compare equal numbers of trials during BCI versus joystick control, in each analyzed session we identified the maximum number of successful trials to each target common to both BCI control and joystick control. For Monkey P, comparing across cortical areas also required analyzing two different sessions, one in which premotor areas had been recorded and another in which parietal areas had been recorded, and we therefore found the maximum number of successful trials common to both tasks in both sessions. We then randomly selected that number of trials for analysis from each type of task in each session, which typically selected all the joystick trials but only ∼30% to 50% of the BCI trials.

Chronically implanted microelectrode arrays often record some of the same units repeatedly in daily sessions over months. To prevent the same unit from being included repeatedly in our analyses, we identified unit recordings that probably originated from the same neuron recorded on different days. The similarity of units recorded from the same electrode was evaluated using four metrics: pairwise cross-correlograms, autocorrelograms, waveform shape, and mean firing rate (Fraser and Schwartz, 2012; Downey et al., 2018). A decision boundary of whether two unit recordings came from the same neuron was drawn using a quadratic classifier under the assumption that the data could be modeled as a mixture of multivariate Gaussians. When two or more units were labeled as having been recorded from the same neuron across days, we retained for the present analyses only the unit with the highest SNR and excluded the others. In this manner 103 of 531 units recorded from Monkey Q and 212 of 823 units from Monkey P were excluded from the following analyses. The left number in each cell of Table 1 gives the number of unique units remaining for analysis in each cortical area for each of the five assignments from monkey Q and three from monkey P.

**Table 1:** Number of units analyzed for each BCI assignment

	Monkey Q						Monkey P			
	i	ii	iii	iv	v	Total	i	ii	iii	Total
BCI units	4 | 4 | 4	4 | 4 | 4	4 | 4 | 2	4 | 4 | 3	4 | 4 | 4	20 | 20 | 17	4 | 4 | 3	4 | 4 | 4	4 | 4 | 2	12 | 12 | 9
Non-BCI										
M1	38 | 30 | 11	30 | 26 | 14	34 | 30 | 15	23 | 20 | 10	35 | 35 | 25	160 | 141 | 75	102 | 87 | 61	125 | 89 | 32	104 | 73 | 29	331 | 249 | 122
PMd	9 | 6 | 4	21 | 18 | 7	12 | 10 | 2	8 | 7 | 5	16 | 15 | 8	66 | 56 | 26	28 | 25 | 18	23 | 17 | 11	30 | 27 | 16	81 | 69 | 45
PMv	23 | 16 | 3	16 | 12 | 3	10 | 8 | 4	17 | 10 | 9	20 | 18 | 10	86 | 64 | 29	23 | 11 | 6	24 | 11 | 3	14 | 9 | 1	61 | 31 | 10
S1	7 | 4 | 4	4 | 2 |0	7 | 3 |1	5 | 2 |0	5 | 4 | 2	28 | 15 | 7	14 | 13 | 8	22 | 16 | 6	18 | 8 | 1	54 | 37 | 15
dPPC	5 | 0 | 0	9 | 5| 3	3 | 2 | 1	11 | 7 | 0	10 | 6 | 2	38 | 20 | 6	15 | 12 | 6	6 | 3 | 1	13 | 3 | 2	34 | 18 | 9
AIP	8 | 5 |3	7 | 4 | 2	5 | 4 | 2	5 | 4 | 3	5 | 5 | 2	30 | 22 | 12	8 | 6 | 2	16 | 9 | 2	14 | 10 | 2	38 | 25 | 6

The three values in each cell give the number of: unique units | units modulated significantly during joystick and/or BCI trials | units modulated significantly during both joystick and BCI trials.

### Task-related modulation

Neuron firing rates in several cortical areas often show cosine tuning as subjects perform a center-out task (Georgopoulos et al., 1982; Kalaska et al., 1983; Prud'homme et al., 1994; Moran and Schwartz, 1999). We therefore considered a unit to be modulated in relation to the center-out task if its firing rate was fit by a classical cosine tuning function:

(4) $$f\left(\theta \right)=\alpha +\beta \times \cos\left(\theta -\theta_{PD}\right)$$

at a significance level of *p *<* *0.05 (*F* test; MATLAB “regress” function). In Equation 4, f(θ) is the firing rate when the peripheral target was centered at θ° (the eight peripheral targets in Fig. 1*D* were assigned to 0° to 315° at 45° intervals); α is the baseline firing rate, β is the absolute modulation depth, and $\theta_{PD}$ is the PD of the unit. Each unit was tested for a cosine fit separately using joystick trials and using BCI trials. The center and right numbers of each cell in Table 1 give the numbers of units significantly cosine-tuned in joystick and/or BCI trials, and in both joystick and BCI trials, respectively.

### PD change (*ΔPD*)

We estimated the *ΔPD* between joystick trials and BCI trials for each unit that was cosine tuned in both tasks by calculating the difference between the unit’s PD during joystick trials ($\theta_{PD\_Joystick}$) and BCI trials ($\theta_{PD\_BCI}$):

(5) $$\Delta PD=\theta_{PD\_BCI}-\theta_{PD\_Joystick}.$$

To determine the statistical significance of each unit’s *ΔPD*, we employed a bootstrap procedure (Chestek et al., 2007). For each unit, we calculated a distribution of PDs by randomly sampling with replacement 1000 times for joystick trials and for BCI trials separately. For each of these two distributions, the mean was subtracted, producing a zero-mean distribution of PDs for joystick trials and for BCI trials. Then a distribution of BCI – joystick *ΔPDs* was gathered by randomly selecting one PD from the zero-mean BCI trial distribution and one from the zero-mean joystick distribution, calculating the difference, and repeating 1000 times, providing a bootstrap *ΔPD* distribution for that unit under the null hypothesis of no BCI – joystick difference (i.e., both with mean = 0). The actual *ΔPD* for that unit then was compared with this bootstrap *ΔPD* distribution. If the actual *ΔPD* was higher than the 97.5th percentile or lower than the 2.5th percentile of the bootstrap *ΔPD* distribution, we considered the unit to have had a significant *ΔPD*. This process was repeated for each unit separately.

We also examined *ΔPDs* at the population level to determine if the entire population rotated to some degree coherently in the same direction resulting in a net change, or whether different units changed in random directions with no net change in the population. If the median of actual *ΔPD*s was significantly different from 0° (circular rank-sum test, circ_medtest function, MATLAB CircStat toolbox; Berens, 2009), we considered that the population had a significant degree of coherent rotation.

### Normalized modulation depth (*nMD*)

To compare the modulation of units with different baseline firing rates, we calculated a *nMD* for each unit that was significantly cosine-tuned during each task separately (joystick or BCI) using the unit’s absolute modulation depth during that task (β; Eq. 4):

(6) $$nMD=\frac{\beta \times 2}{FR^{80\%}-FR^{20\%}}.$$

Unlike the values used for BCI units on-line (Eq. 1), here $FR^{80\%}$ and $FR^{20\%}$are the 80th and 20th percentiles, respectively, of the overall firing rate distribution pooling data from both joystick and BCI trials. This *nMD* for either joystick trials or BCI trials therefore could be >1 if the unit was modulated intensely in one of the tasks and had relatively low firing rates during the other. In general, the higher the *nMD*, the more intensely the unit was modulated.

### Effective connectivity analysis

To examine effective connectivity among simultaneously recorded units, we used Granger causality adapted for point processes (Kim et al., 2011; Balasubramanian et al., 2017). This adaptation replaces the standard multivariate vector autoregressive models with point process likelihood functions, where the point process of a spike train is characterized by the logarithm of a conditional intensity function (CIF) modeled with a generalized linear model (GLM). To optimize both the temporal resolution of the models and the ability to detect effective connectivity at various latencies, CIFs were calculated with durations from 3 to 99 ms in 3 ms steps, and the resulting GLM that provided the best approximation of the spike trains was selected using Akaike’s information criterion (AIC). The optimal spike histories determined in this way had a median duration of 12 ms and 90th percentile of 51 ms.

For each unit, *i*, the point process likelihood then was modeled first with covariates including the spiking history of that unit and all other simultaneously recorded units, characterized by a parameter vector, $\gamma_{i}$, and second with the same covariates but excluding the spiking history of another unit, *j*, characterized by parameter vector denoted, $\gamma_{i}^{j}$. The log-likelihood ratio for these two models then is, Γ*_ij_*:

(7) $${\text{Γ}}_{ij}=\log\frac{L_{i}(\gamma_{i}^{j})}{L_{i}(\gamma_{i})}.$$

Because excluding the information provided by unit *j* can only degrade the modeling of unit *i* and decrease the likelihood $L_{i}(\gamma_{i}^{j})$ relative to $L_{i}(\gamma_{i})$, the ratio of likelihoods is always ≤1 and the log likelihood ratio is always ≤0, theoretically being = 0 if unit *j* has no effect on unit *i* and increasingly <0 the stronger the effect. A Granger causality measure (GCM), $\Phi_{ij}$, then can be calculated as:

(8) $$\Phi_{ij}=-sign\left(\gamma_{i}^{j}\right){\text{Γ}}_{ij},$$

where positive values indicate an excitatory effect of unit *j* on unit *i* and negative values indicate an inhibitory effect.

This analysis was performed using all *Q* simultaneously recorded spike trains from 750 ms before to 2250 ms after the Go cue in each analyzed trial, providing a *Q* × *Q* matrix of GCMs, the $\Phi_{ij}$, i.e., the strength of the effect of *j^th^ unit* on the *i^th^* unit. Significance testing was performed on the ${\text{Γ}}_{ij}$, the distribution of which approaches the ${\text{χ}}^{2}$ distribution for large *Q*. The Benjamini–Hochberg procedure was applied to control the false discovery rate at 0.05. The Granger connectivity of each unit *j* to unit *i* then was classified as (1) no significant connectivity, (2) significant excitatory connectivity, or (3) significant inhibitory connectivity.

We performed a simulation to examine the extent that identifying effective connectivity in this way would be influenced by the modulation depth of the unit pair being tested. We simulated a population of 30 units: 15 units with a relatively low *nMD* of 0.35 and 15 with a relatively high *nMD* of 0.75. For each unit, we generated spike trains in 1 ms bins for 10 trials to each of the eight targets, all lasting 1 s. Each unit was assigned a randomly generated PD with a baseline firing rate, *FR_baseline_*, drawn from a uniform distribution in the range [10, 30] Hz. The average *FR_baseline_* thus was 20 Hz for both low *nMD* and high *nMD* populations. Spikes of unit *i* for a simulated trial with a target located at θ° were generated using a commonly used procedure (Koch, 2004; Kim et al., 2011). At each 1 ms time step, a random number uniformly distributed in the range [0, 1] was generated and compared with a threshold, $\delta_{\theta }^{i}$, based on cosine directional tuning with the unit’s baseline firing rate ($FR_{baseline}^{i})$, modulation depth ($MD^{i}$), and PD ($\theta_{PD}^{i})$:

(9) $$\delta_{\theta }^{i}=\frac{1000}{FR_{baseline}^{i}+MD^{i}\times \text{cos}(\theta -\theta_{PD}^{i})}.$$

If the randomly generated number was smaller than $\delta_{\theta }^{i}$, a spike was simulated to occur in that 1 ms bin unless a spike had occurred in the one preceding bin (1 ms refractory period).

Within this population of 30 neurons, we created 12 artificial connections: three excitatory connections and three inhibitory connections from a low *nMD* unit to another low *nMD* unit, and three excitatory connections and three inhibitory connections from a high *nMD* unit to another high *nMD* unit. The target unit of each excitatory (or inhibitory) connection was assigned a 50% higher (or lower) likelihood of firing a spike at time *t* if the trigger unit had fired a spike at any time in the range from 10 to 6 ms before *t*. We then ran 1000 simulations using the methods described above (Eqs. 7, 8), for each simulation seeding the population with re-randomized $FR_{baseline}^{i}$ and $\theta_{PD}^{i}$. All 12 artificial connections were detected in every simulation. Of the 858 potential false positive connections tested for which no artificial connection was present (858 = 30^2^ potential connections, minus 30 self-connections, minus 12 true positives), no more than 13 false positives were detected (13/858 = 0.015) in any of one the 1000 simulations, with an average false positive rate of 0.0021 across the 1000 simulations, all within our accepted false discovery rate of 0.05. Furthermore, the false positive rate among the low-low *nMD* pairs averaged across simulations was 0.0020; among low-high pairs, 0.0019; among high-low pairs, 0.0021; and among high-high pairs 0.0025. These false positive rates were significantly different among the four connection groups (*p *<* *0.005, Kruskal–Wallis tests), because of a higher false positive rate among high-high pairs compared with low-low pairs (*p *<* *0.005, *post hoc* Kruskal–Wallis tests with Bonferroni correction). Although the difference of ∼0.0005 (five false positives in 10,000 tests) between low-low pairs and high-high pairs might have contributed an extra high-high false positive connection to the populations tested (∼1000 tests per population) in Results below, such a small difference would not have altered any of the findings described.

## Results

### Behavioral performance during joystick and BCI trials

In each session, the monkey performed the center-out task first using the joystick and then the BCI, both within ∼2 h. Monkey Q was trained to proficient BCI performance, sessions with ≥400 successfully performed BCI trials at a success rate ≥80%, with five different BCI-unit assignments, Monkey P with three. For each assignment, we selected for the present analyses a session recorded once the monkey had been performing at this level consistently. For Monkey Q the selected sessions were recorded 16, 9, 10, 7, and 10 d after training with a new BCI-unit assignment began; for Monkey P, in which two sessions were required to record from all cortical areas, after 16/17, 10/11, and 13/14 d. Across these analyzed sessions, monkey Q performed at a higher success rate during BCI trials than during joystick trials (BCI, 94.7 ± 1.4%; joystick, 76.6 ± 3.6%; *p* < 1e-16, χ^2^ test), whereas Monkey P’s success rates were similar (BCI, 85.7 ± 5%; joystick, 86.5 ± 5.7%; *p *=* *0.32, χ^2^ test). Although Monkey P performed at a higher success rate than Monkey Q during joystick trials (*p *< 5e-12, χ^2^ test), Monkey Q performed at a higher success rate than Monkey P during BCI trials (*p *< 1e-16).

**Figure 4.**
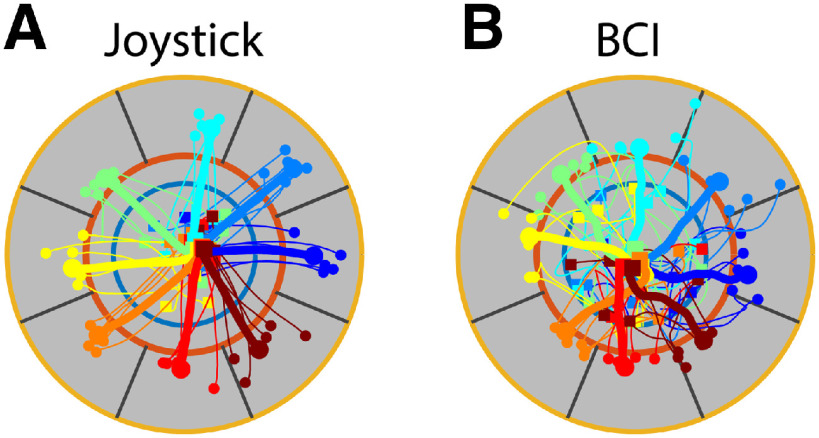
Cursor paths in joystick trials (***A***) and BCI trials (***B***). Each trial started within the center target (blue circle) and ended in one of the eight peripheral targets (between the red and yellow circles). Thin lines show the cursor paths to each target (colors blue to brown) from five successful trials, and thick lines represent cursor paths averaged across 20 successful trials to each target (time normalized between Go Cue and Success events). Squares and solid dots show the cursor positions at the time of Go Cue and Success, respectively. Data from Monkey Q, assignment iii.

Although both monkeys met our criteria for proficient BCI control during these analyzed sessions, their performance in BCI trials was not equivalent to that in joystick trials. As detailed in Materials and Methods, because our arbitrary assignment of BCI units to the four cardinal directions made learning to use each decoder quite challenging, we relaxed the criteria for successful performance of BCI trials as compared with joystick trials. Figure 4 compares the cursor trajectories of five successful joystick trials and five successful BCI trials (thin lines) involving each of the eight targets, as well as the average over 20 trials each (thick lines), all from the same session. Whereas the cursor moved in relatively straight paths in individual joystick trials, in individual BCI trials the trajectories often were convoluted, although on average directed to the target.

We quantified these differences by measuring the path length and the response time, both from the time of the Go cue to the Success, in all analyzed trials. Pooling across the analyzed sessions from each monkey, path lengths were longer during BCI trials in both monkeys [Monkey Q, joystick, 486 ± 104 screen units (mean ± SD), BCI, 755 ± 348 screen units, *p* < 5e-44, Wilcoxon rank-sum test; Monkey P, joystick, 507 ± 110 screen units, BCI, 863 ± 423 screen units, *p* < 5e-47]. Response times also were longer in BCI trials for Monkey P (joystick, 1.31 ± 0.20 s; BCI, 2.20 ± 1.19 s; *p *< 5e-29, Wilcoxon rank-sum test), although not for Monkey Q (joystick, 1.44 ± 0.33 s; BCI, 1.66 ± 0.84 s, *p* = 0.16). These performance measures also showed additional differences between monkeys. During joystick trials Monkey P had shorter paths and response times than Monkey Q (path lengths: *p* < 5e-3, response times: *p* < 1e-9, Wilcoxon rank-sum tests), while during BCI trials Monkey Q had shorter paths and response times than Monkey P (path lengths: *p* < 5e-4, response times: *p* < 5e-11). Although Monkey P’s joystick performance was superior in every measure to that of Monkey Q, Monkey Q’s BCI performance was superior to that of Monkey P.

### Neurons in multiple cortical areas were modulated during both joystick and BCI trials

**Figure 5.**
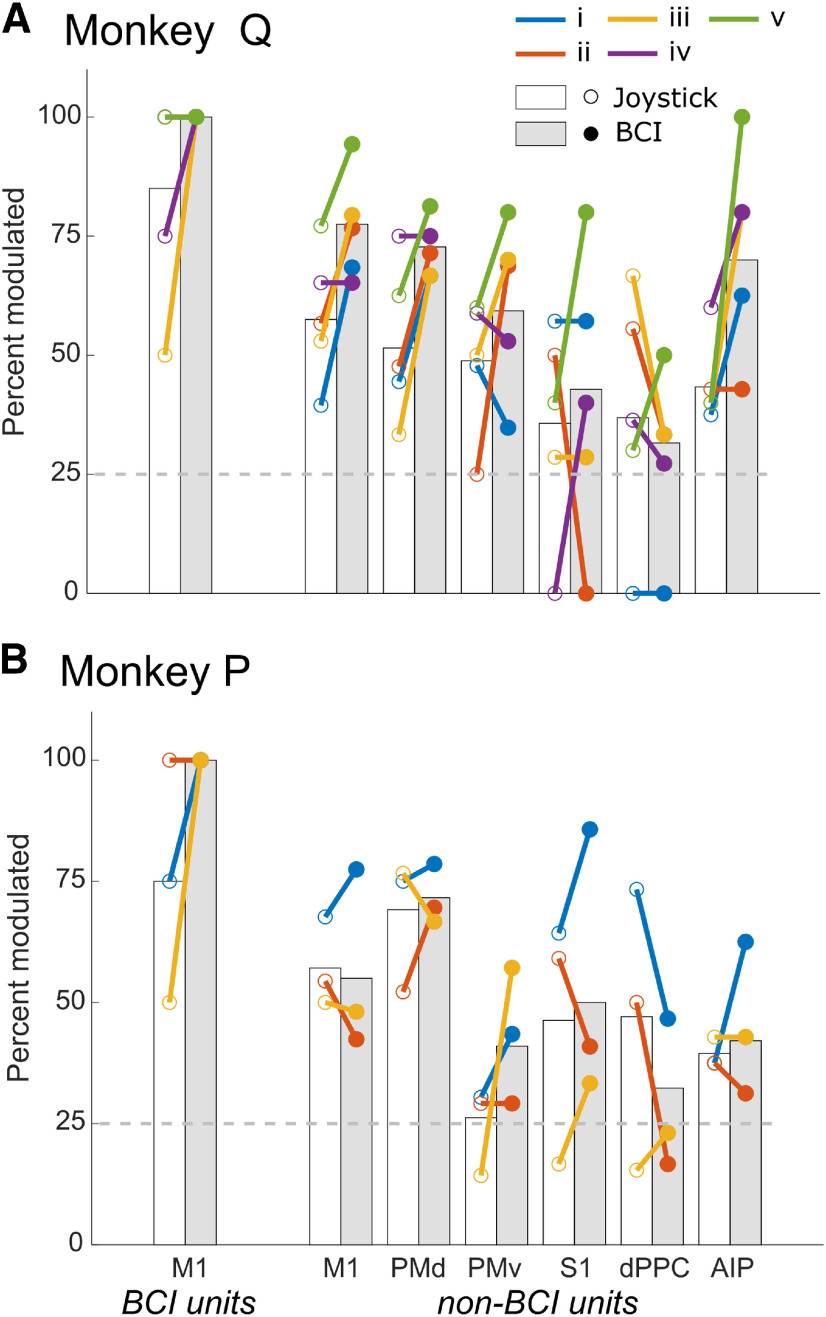
Percent of units modulated significantly with the center-out task during joystick trials and during BCI trials. ***A***, Monkey Q. ***B***, Monkey P. Bars represent the percentages of units across all assignments during joystick trials (white) or BCI trials (gray), while colored lines compare percentages during joystick trials (open circles) versus BCI trials (filled circles) in individual BCI assignments. Because all BCI M1 units were modulated during the BCI task, closed circles overlap for those units at 100%. Open circles and colored lines also overlapped at 100% for BCI units during joystick trials for Monkey Q in assignments i and ii.

We tested each analyzed unit for cosine tuning separately during joystick trials and during BCI trials, using the unit’s firing rate averaged from the Go cue to Success in each analyzed trial. The leftmost pair of bars in Figure 5 shows the percent of BCI units and the other pairs of bars the percent of non-BCI units in each cortical area that were cosine tuned during the joystick task (white) and during the BCI task (gray) averaged across all BCI-unit assignments in each monkey. Colored circles with connecting lines show the percentages in each assignment separately. With a few exceptions, 25% (arbitrarily chosen level, dashed horizontal line) or more of the sorted units in each cortical area were modulated during both tasks in each assignment. Task-related modulation of non-BCI units thus was common in all six cortical areas not only during joystick control of the cursor, but also during BCI control.

On average, 75–80% of the BCI units in each monkey were modulated significantly during joystick trials (Fig. 5, BCI units, white bar), whereas during BCI trials 100% of the BCI units were modulated significantly for every BCI assignment in both monkeys (Fig. 5, BCI units, gray bar). Did the percentage of modulated non-BCI units also increase during BCI control? In monkey Q, this percentage tended to increase during BCI control as compared with joystick control. This increase was significant when non-BCI units were pooled across sessions and across all 6 cortical areas (McNemar’s test, *p < *1e-7). *Post hoc* pairwise testing for individual cortical areas showed significant increases among non-BCI units in M1, PMd, and AIP (McNemar’s test with Bonferroni correction, *p *<* *0.0083 = 0.05/6 cortical areas). Monkey P, however, did not show any significant changes in the percentage of non-BCI units modulated during BCI control versus joystick control. Monkey Q but not Monkey P thus had more modulated non-BCI units in some cortical areas during BCI control than during joystick control. In monkey Q, this increase in modulated units may have contributed to superior performance during BCI trials as compared with Monkey P.

### Non-BCI units in multiple cortical areas changed PD during BCI trials

**Figure 6.**
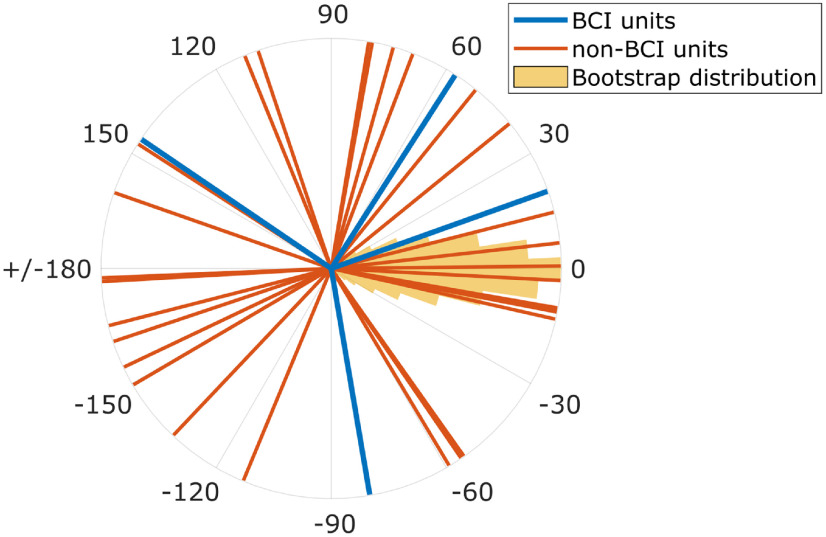
*ΔPD*s in a single session. Lines indicate the Δ*PD* of the four BCI units (blue) and 29 non-BCI units (red) in polar coordinates. The yellow histogram represents the bootstrapped probability distribution of Δ*PDs* under the null hypothesis of no change. All four BCI units, as well as 72% of the non-BCI units, had a significant *ΔPD* during BCI trials compared with joystick trials. At the population level, the Δ*PD *among non-BCI units was not significantly different from 0°. Data from Monkey Q, BCI assignment ii.

Given that the firing rate of most of BCI units and many non-BCI units modulated with both joystick and BCI trials, we compared the PD of individual units that were significantly cosine-tuned during both tasks (Table 1, right number in each cell). Figure 6 shows, for example, the change in PD (*ΔPD = PD_BCI_* – *PD_Joystick_*) for the four BCI units (blue) and the 29 non-BCI M1 units (red) from a single session, along with the bootstrapped distribution of the combined population (BCI and non-BCI units) expected if there were no changes in PDs.

All four BCI units in this illustrated session had a significant *ΔPD* between joystick and BCI trials. Across all assignments, only one of the 26 BCI units that were tuned during both tasks did not show a significant change in the PD between joystick and BCI trials. Did the PDs of BCI units change toward the cardinal directions to which they had been assigned arbitrarily by the decoder? Knowing the direction assigned to each BCI unit, we calculated the absolute value of the difference between that assigned direction and the PD of each BCI unit during joystick trials and again during BCI trials. Across all 26 testable BCI units, this absolute assigned-preferred direction difference was significantly smaller for BCI than for joystick trials (Monkey Q, *p* < 5e-5; Monkey P, *p* < 0.05, Wilcoxon rank-sum tests). As might have been expected, therefore, the PDs of BCI units on average changed toward their assigned directions.

Of the 29 non-BCI units illustrated in Figure 6, the PDs of 21 (72%) also changed significantly. And pooling across assignments, the majority of the non-BCI units in each cortical area in each monkey showed significant changes in PD with the exceptions of dPPC and AIP in Monkey Q (Table 2). Changes in PD thus were common among non-BCI units in both monkeys.

**Table 2:** Percent of units with a significant joystick versus BCI change in PD

	BCI units	Non-BCI	M1	PMd	PMv	S1	dPPC	AIP	All
Monkey Q	100%		84%	81%	52%	86%	33%	42%	72%
Monkey P	89%		77%	67%	60%	87%	78%	67%	74%

Systematic changes in PD at the population level can provide insight into the strategy used to perform a BCI task. In particular, an average *ΔPD* significantly different from 0° suggests a re-aiming strategy in that most PDs are shifted in the same direction. Re-aiming may provide a useful strategy when the directions assigned to BCI units on average constitute a relatively consistent rotation of their natural PDs. The four BCI units used here in a given assignment were too few to assess consistent rotation statistically, however. We therefore classified each BCI unit with a significant *ΔPD* as having a *ΔPD *<* *180°, *ΔPD* = 180°, or *ΔPD* > 180°. Five of the eight BCI-unit assignments included at least one BCI unit with *ΔPD *<* *180° and at least one with *ΔPD *>* *180°, making a re-aiming strategy unlikely.

For populations of non-BCI units pooled across all cortical areas or within individual cortical areas, a median *ΔPD* significantly different from 0° was found in only one of the eight assignments (Qiii, circular rank-sum test, *p < *0.05/8 assignments). Significant differences from 0° were not found in any of the individual cortical areas for any of the BCI-unit assignments. The *ΔPDs* of individual non-BCI units thus occurred in various directions and amplitudes, resulting in no net *ΔPD* for any population. Considering the non-BCI units as a surrogate indicator, these findings suggest that, except perhaps in one assignment, the directions assigned to BCI units did not provide a consistent rotation of their natural PDs as assessed during joystick trials.

### *nMD* increased in some cortical areas during BCI control

**Figure 7.**
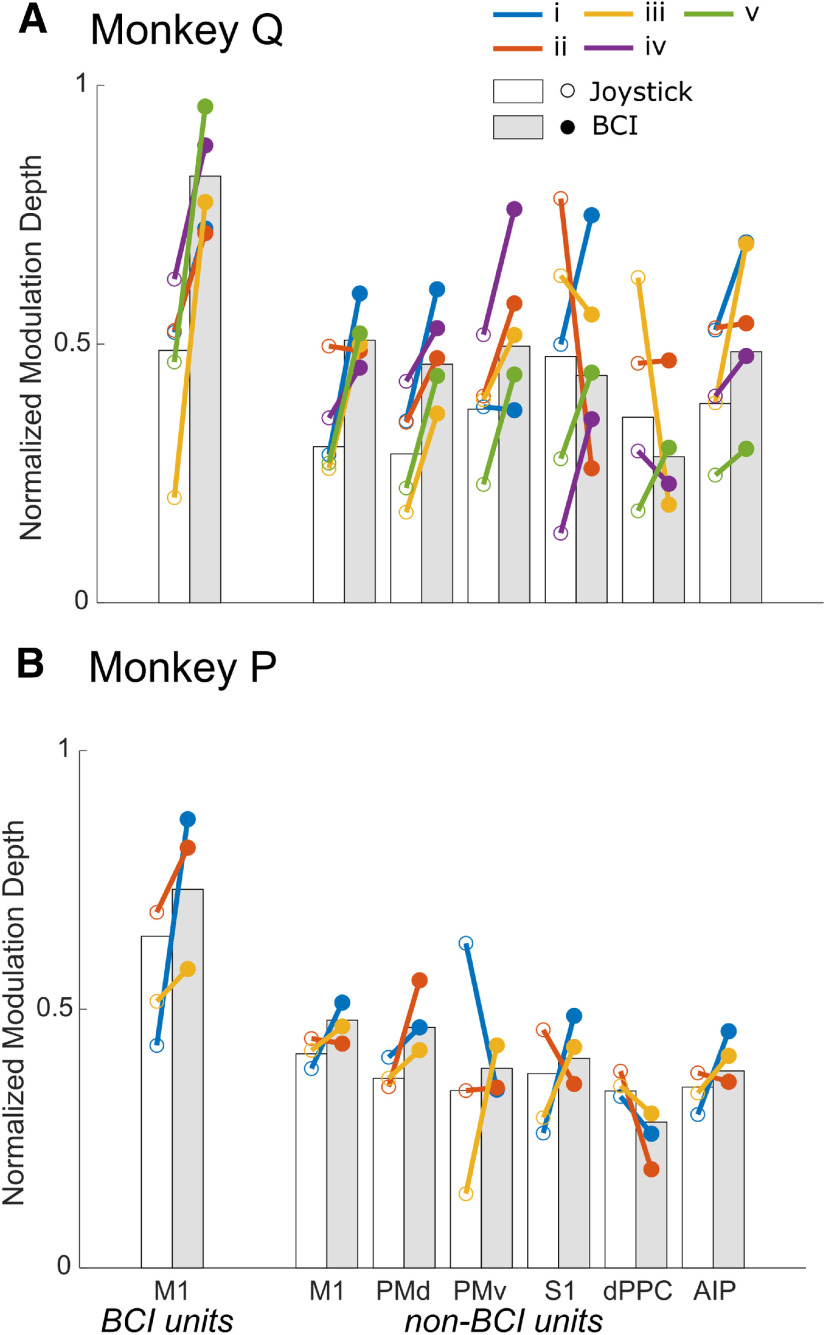
*nMD* of significantly modulated units during joystick trials versus BCI trials. ***A***, Monkey Q. ***B***, Monkey P. Bars represent the median *nMD* among units across all assignments during joystick trials (white) or BCI trials (gray), while colored lines compare median *nMDs* during joystick trials (open circles) versus BCI trials (filled circles) in individual BCI assignments.

As might have been expected of the BCI units, all of which became modulated during BCI trials, their average modulation depth also increased during BCI trials as compared with joystick trials in each monkey. As illustrated in Figure 7, left, this increase in average *nMD* among BCI units occurred consistently in all sessions in both monkeys. At the level of individual units, *nMD* increased in 17 of the 20 BCI units in Monkey Q and eight of 12 units in Monkey P during BCI as compared with joystick trials. Similarly, among non-BCI units *nMD* often increased during BCI trials, although less consistently than among BCI units. Figure 7 shows that in each cortical area except dPPC, the median *nMD* of non-BCI units increased during BCI trials in three or more of the five assignments for Monkey Q, and in two or more of the three assignments for Monkey P. We therefore compared *nMD* during joystick versus BCI trials for non-BCI units in all six cortical areas. For this comparison, we included units that showed significant modulation during either joystick or BCI trials, or both (Table 1, center number in each cell). Pooling across all assignments and cortical areas, the median *nMD* of non-BCI units was greater during BCI trials than during joystick trials in monkey Q, although the trend fell short of significance in Monkey P (Monkey Q, *p *<* *5e-11; Monkey P, *p *=* *0.058; Wilcoxon signed-rank tests). *Post hoc* testing for each cortical area separately (Fig. 7, gray vs white bars) showed significant increases in M1, PMd, and PMv in Monkey Q (Wilcoxon signed-rank test with Bonferroni correction, *p *<* *0.05/6). As for the increase in the percentage of non-BCI units that were modulated during BCI trials in Monkey Q, this increase in modulation depth may have contributed to superior performance during BCI trials as compared with Monkey P.

Modulation depth also varied to some degree among cortical areas. Pooling the data across assignments we found no significant differences in *nMD* among cortical areas during joystick trials in either monkey (Monkey Q, *p = 0.73;* Monkey P, *p = 0.09*; Kruskal–Wallis tests). During BCI trials, however, the variation in *nMD* among cortical areas was significant in each monkey (Monkey Q, *p *<* *5e-3; Monkey P, *p *<* *1e-3, Kruskal–Wallis tests). *Post hoc* pairwise comparisons revealed that in Monkey Q the median *nMD* of non-BCI units in M1 or in PMv was significantly larger than in dPPC, and in Monkey P the median *nMD* of non-BCI units in M1 or in PMd was larger than in dPPC (*p *<* *0.0083 = 0.05/6, Kruskal–Wallis tests with Bonferroni correction for six cortical areas). Differences in *nMD* between cortical areas thus appeared during BCI trials that were not present during joystick trials. Modulation in M1 and premotor areas became larger than that in dPPC, which may have reflected increased modulation in frontal motor areas and/or a decrease in proprioceptive feedback to dPPC during BCI trials as compared with joystick trials.

### Effective connectivity during joystick and BCI trials

**Figure 8.**
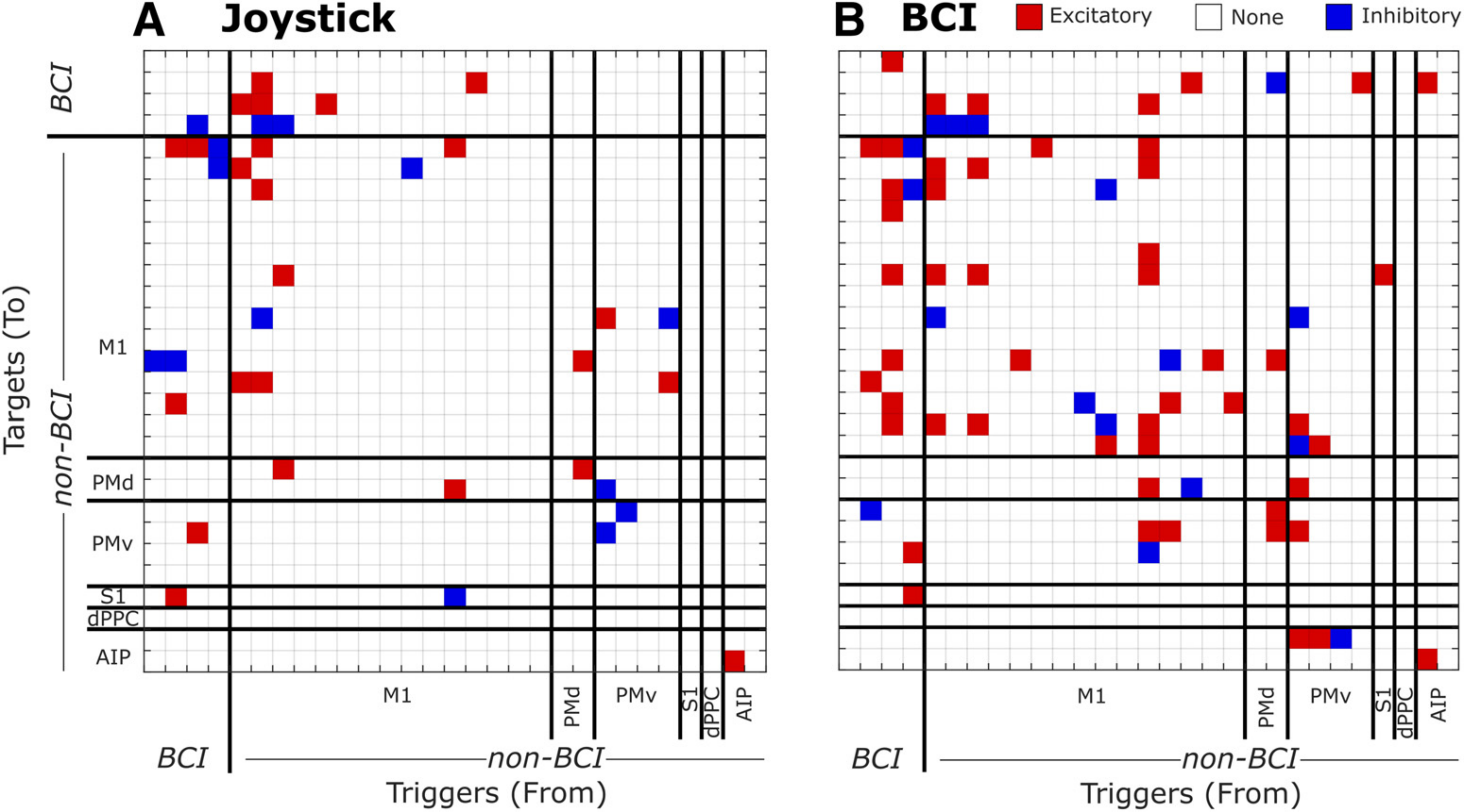
Granger connectivity matrices in a single session. Granger connectivity was evaluated separately during joystick trials (***A***) and during BCI trials (***B***). Each red or blue cell indicates significant excitatory or inhibitory connectivity from a trigger unit (abscissa) to a target unit (ordinate). White cells indicate no significant connectivity between the pair. Black lines separate groups of BCI units and non-BCI units from different cortical areas. Data from Monkey Q, assignment iii.

The changes observed in the percent of units modulated, their PDs, and their *nMD*s suggest that non-BCI units in multiple cortical areas participated indirectly in controlling the BCI, either influencing the activity of the BCI units, or being influenced by the BCI units, or both. We therefore compared effective connectivity among both BCI and non-BCI units during joystick trials and during BCI trials. We evaluated pairwise effective connectivity among all BCI units (Table 1, top row, left number) and all non-BCI units that were modulated significantly during both joystick and BCI trials (Table 1, other rows, right number in each cell) using Granger causality adapted for point process models (Kim et al., 2011; Balasubramanian et al., 2017), as described in Materials and Methods. Figure 8 shows the Granger connectivity matrices from an example session, computed using equal numbers of joystick (Fig. 8*A*) and BCI (Fig. 8*B*) trials. Among the 29 simultaneous recorded units, pairs with excitatory effective connectivity from the trigger unit (abscissa) to the target unit (ordinate) are indicated by a red square and those with inhibitory connectivity by a dark blue square.

Comparing effective connectivity during joystick versus BCI trials shows that changes occurred both within and between cortical areas, and both with BCI and with non-BCI units. For the example session shown in Figure 8, the number of connected pairs increased in many instances, for example, from the BCI units to non-BCI units in M1, from non-BCI units in M1 to other non-BCI units in M1, and from non-BCI M1 units to those in PMv, while decreasing in others, for example from PMv units to other PMv units). Overall, the fraction of significantly connected pairs in this session was greater during BCI than during joystick trials.

**Figure 9.**
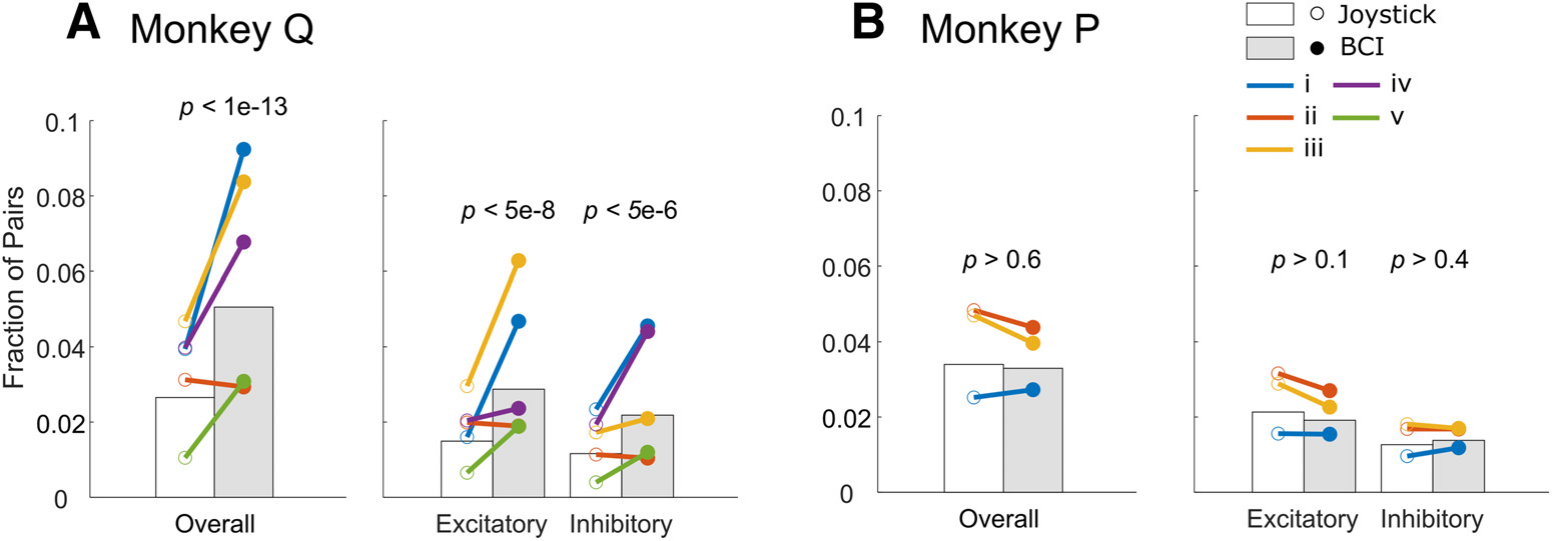
The fraction of unit pairs with significant effective connectivity during joystick-controlled trials (white bars) and BCI-controlled trials (gray bars) for Monkey Q (***A***) and Monkey P (***B***). For each monkey, overall fractions are shown to the left and the fractions with excitatory or inhibitory connectivity are shown separately to the right. White and gray bars represent fractions pooled for each monkey across assignments (with *p* values from McNemar’s tests), while colored lines represent the individual BCI-unit assignments.

Pooling across sessions and across cortical areas in Monkey Q, the fraction of unit pairs with effective connectivity was significantly larger during BCI trials (Fig. 9*A*, left, gray bar) than during joystick trials (Fig. 9*A*, left, white bar) in Monkey Q (*p *<* *1e-13, McNemar’s test) but not in Monkey P (Fig. 9*B*, left, *p *>* *0.6). The increase during BCI trials remained evident in Monkey Q when excitatory (*p *<* *5e-8) and inhibitory (*p *<* *5e-6) connections were considered separately (Fig. 9*A*, right), but the fraction of neither excitatory (*p *>* *0.1) nor inhibitory (*p *>* *0.4) connections changed in Monkey P (Fig. 9*B*, right). In Monkey Q, this increase in effective connectivity during BCI trials was present in four of the five individual assignments (Fig. 9*A*, colored lines). Again, increased effective connectivity may have contributed to Monkey Q’s superior performance during BCI trials as compared with Monkey P.

### Non-BCI units with effective connectivity to or from a BCI unit

Non-BCI units with effective connectivity either to or from a BCI unit might influence BCI control more strongly than those without such connections. An increase of modulation depth in a non-BCI unit with effective connectivity to a BCI unit, for example, might have a greater impact on that BCI unit than the same increase in a non-BCI unit without such connectivity. We therefore divided the non-BCI units into two groups: Connect+ units had significant excitatory or inhibitory connectivity, either as a trigger or as a target, with at least one BCI unit; Connect– units had no significant effective connectivity with any of the BCI units, although Connect– units might or might not have effective connectivity with other non-BCI units. Table 3 gives the percentages of Connect+ units in each cortical area, pooled across assignments in each monkey, for both joystick and BCI trials, and further subdivides the Connect+ units into those being the trigger for versus the target of connectivity with a BCI unit.

**Table 3:** Percentages of Connect+ non-BCI units

Monkey	Task	Non-BCI units	M1	PMd	PMv	S1	dPPC	AIP	Overall
Monkey Q	Joystick	Connect+	27	12	14	57	17	8	21
		Connect+_trigger_	20	4	4	14	0	8	13
		Connect+_target_	13	8	10	43	17	8	13
	BCI	Connect+	36	42	38	43	0	25	36
		Connect+_trigger_	23	27	21	14	0	17	21
		Connect+_target_	32	35	21	43	0	8	28
Monkey P	Joystick	Connect+	30	20	30	27	22	17	27
		Connect+_trigger_	18	11	20	13	0	17	15
		Connect+_target_	19	11	20	20	22	0	17
	BCI	Connect+	38	36	20	40	0	33	35
		Connect+_trigger_	23	20	10	7	0	33	20
		Connect+_target_	25	20	10	33	0	0	22

In both monkeys, pooling across cortical areas revealed an overall increase in the percent of Connect+ units during BCI trials as compared with joystick trials (Monkey Q, 36% vs 21%, *p *<* *0.005; Monkey P, 35% vs 27%, *p < *0.05, McNemar’s test). *Post hoc* pairwise testing, however, found no significant differences in any of the 6 individual cortical areas in either monkey (McNemar’s tests with Bonferroni correction for six tests, *p *>* *0.05/6). Nor did the percent of Connect+ units differ significantly among cortical areas in either monkey during joystick trials (Monkey Q, *p *>* *0.05; Monkey P, *p *>* *0.8, χ^2^ tests) or during BCI trials (Monkey Q, *p *>* *0.4; Monkey P, *p *>* *0.2, χ^2^ tests). Although not demonstrable for individual cortical areas, in both monkeys the overall percentage of non-BCI units with effective connectivity to or from BCI units increased during BCI as compared with joystick control.

Non-BCI units providing effective connections to BCI units might play a different role than those receiving effective connections from BCI units. We therefore classified Connect+ non-BCI units into those with effective connectivity to a BCI unit (Connect+_trigger_) and those with effective connectivity from a BCI unit (Connect+_target_). Note that these classes are not mutually exclusive because, although each unit pair can have only one effective connection, the same non-BCI unit could have an effective connection to one BCI unit and receive an effective connection from another BCI unit. Percentages of Connect+_trigger_ and Connect+_target_ units are given in Table 3, pooling across assignments for each monkey. When pooling across cortical areas in each animal separately, the percentage of Connect+_trigger_ units was not significantly different from the percentage of Connect+_target_ units either during joystick trials or during BCI trials (*p *>* *0.05, McNemar’s tests). Nor were significant differences found for any of the individual cortical areas during either joystick or BCI trials (McNemar’s test with Bonferroni correction for six tests, *p *>* *0.05/6). Thus, similar percentages of non-BCI units in each cortical area provided effective connections to and received effective connections from BCI units during each task.

**Figure 10.**
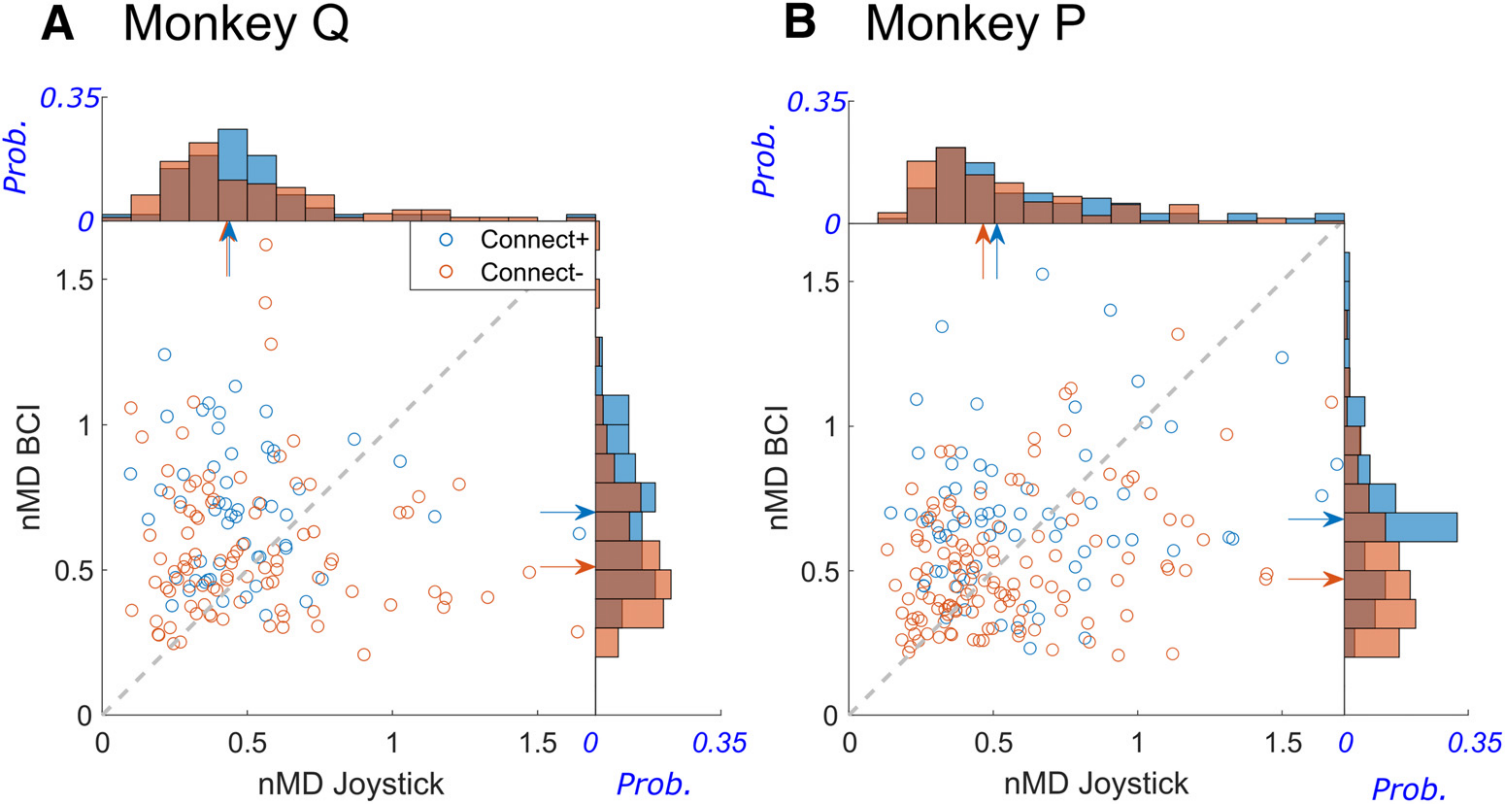
*nMD*s of Connect+ units and Connect– units compared during joystick versus BCI trials. ***A***, Monkey Q. ***B***, Monkey P. Scatter plots show that the *nMDs* of Connect+ units (blue circles) were higher during BCI trials, as the majority of blue circles fall above the dashed line of unity slope. The same was not true for Connect– units (orange circles). Marginal histograms show the probability distributions of Connect+ *nMDs* (blue) and Connect– *nMD*s (orange) during BCI trials (right) which were significantly different, and during joystick trials (top) which were not. Blue and orange arrows represent the medians for Connect+ and Connect– unit populations, respectively. In these scatterplots, outliers with *nMD *>* *1.7 have been excluded for purposes of display; including the outliers did not change the results.

As described above, we found that the *nMD* of most BCI units was greater during BCI trials than during joystick trials, as was the *nMD* of many non-BCI units. Did this increase in *nMD* during BCI trials occur selectively in Connect+ units as compared with Connect– units? Figure 10 shows a scatterplot for each monkey in which each point represents the *nMD* for a non-BCI unit during joystick (abscissa) versus BCI (ordinate) trials, along with the respective marginal probability distributions. Colors distinguish Connect+ (blue) versus Connect– (orange) units pooled from all assignments in each monkey. More evident in Monkey Q than in Monkey P is that the majority of Connect+ units lie above the dashed line of unity slope, indicating that on average Connect+ units had a larger *nMD* during BCI trials than during joystick trials, which was the case in both monkeys (Monkey Q, *p < *5e-6; Monkey P, *p < *0.05, Wilcoxon signed-rank tests). In contrast, the population of Connect– neurons showed no systematic joystick versus BCI difference in *nMD* in either monkey (Monkey Q, *p *>* *0.05; Monkey P, *p *>* *0.9, Wilcoxon signed-rank tests). The marginal probability distributions confirm this difference between Connect+ and Connect– neurons. During BCI trials, the median *nMD* of Connect+ units was greater than that of Connect– units in both monkeys (right histograms, Monkey Q, *p < *5e-4; Monkey P, *p < *5e-7, Wilcoxon rank-sum tests) whereas during Joystick trials the median *nMD* of Connect+ and Connect– units was not different (top histograms, Monkey Q, *p > *0.8; Monkey P, *p > *0.1, Wilcoxon rank-sum tests). While the scatterplots of Figure 10 exclude outliers with *nMD *>* *1.7 (six units from Monkey Q, nine from Monkey P) for purposes of display, including all outliers did not change any of these findings. The modulation depth of Connect+ neurons thus increased during BCI trials while that of Connect– neurons did not.

## Discussion

The firing rates of neurons in multiple cortical areas are modulated during voluntary upper extremity movements. We found that while only four M1 units controlled a closed-loop BCI, substantial numbers of non-BCI units not only in frontal motor areas (M1, PMd, PMv) but also in parietal areas (S1, dPPC, AIP) likewise were modulated in relation to the task. In each of these cortical areas, generally comparable percentages of non-BCI units were modulated during joystick and BCI control, although in Monkey Q the percentage was significantly higher during BCI trials in M1, PMd, and AIP. Many non-BCI units were cosine-tuned during both joystick control and BCI control of the cursor, and among these units we found changes in PD, modulation depth, and effective connectivity during BCI trials as compared with joystick trials, similar to the changes that occurred concurrently in the BCI units. All these cortical areas thus participate not only in natural control of voluntary limb movement but in a more general system for closed-loop control of an effector being moved to a visual target. And many individual non-BCI neurons in these areas change their activity between joystick and BCI control.

Our findings have two important limitations. First, whereas the joystick-controlled movements typically had a large rapid and relatively ballistic initial component that often brought the cursor promptly to the target, the BCI-controlled movements were generally slower and less smooth, often requiring multiple corrective sub-movements before arriving in the target (Fig. 4). This difference was particularly evident because we chose to use a more permissive center-out task for BCI-controlled trials and chose not to train the monkeys to the point of BCI performance equivalent to that with joystick control. Some of our findings may have resulted from these differences between the present joystick-controlled versus BCI-controlled trials. The latter might be viewed as a comparatively early stage in learning to use a difficult, non-intuitive controller.

Second, rather than focusing our recording channel capacity on one or two cortical areas, for the present study we distributed the available channels across six cortical areas. Fewer units therefore were recorded from any one area than in some other studies. Furthermore, across BCI-assignments in a given monkey and across monkeys, our microelectrode arrays consistently recorded fewer analyzable units in parietal than in frontal cortical areas (Table 1). The number of units recorded may have limited the present statistical comparisons among areas. Had we recorded more units, particularly in S1, dPPC, and AIP, additional significant differences might have emerged. Nevertheless, we have included these parietal areas along with the frontal areas in the present analyses as providing our best available sampling of their activity.

### Strategies and cognitive load elicited by the BCI decoder

For the present study, we intentionally chose a non-intuitive BCI decoder to require a relatively high cognitive load, potentially eliciting more extensive activity in the various cortical areas from which we recorded. Because using fewer units tends to make BCI control less accurate (Law et al., 2014), we limited the number of BCI units to four, and assigned each BCI unit arbitrarily to drive cursor velocity in a particular cardinal direction without regard to its PD during joystick trials. Under such conditions BCI control can be achieved through a variety of strategies, including remapping the PDs of individual BCI units, reweighting their modulation depths, and re-aiming with a coherent rotation of the PDs of most units (Jarosiewicz et al., 2008; Ganguly et al., 2011; Chase et al., 2012; Sakellaridi et al., 2019). Across the eight assignments in the two monkeys, PDs changed significantly in all but one of the BCI units that were also modulated during the joystick task. On average the PD of each BCI unit shifted toward its own assigned direction, with some changing in clockwise and others in counterclockwise directions in five of the eight assignments. In addition, the average modulation depth of the BCI units increased in every assignment. In only one of the eight assignments did the population of non-BCI units show evidence of a coherent rotation all in the same direction. Whereas remapping and reweighting thus occurred in most assignments, re-aiming was uncommon.

Our BCI decoder design was sufficiently difficult for the monkeys to use that we relaxed our criteria for successful trial performance compared with joystick trials. Even after several days of BCI training when the monkeys had achieved our criteria of >80% success over at least 400 trials, cursor trajectories remained considerably longer and more convoluted during BCI trials than during joystick trials, and non-BCI units remained as deeply modulated during BCI trials as during joystick trials (Hwang et al., 2013; Law et al., 2014). Had we used a BCI decoder optimized to incorporate the natural tuning of larger numbers of M1 neurons (Jarosiewicz et al., 2008; Ganguly and Carmena, 2009; Gilja et al., 2012; Sadtler et al., 2014) and/or trained the monkeys for substantially more sessions (Oby et al., 2019; Zhou et al., 2019), the response time and path length of BCI trials might have more closely approximated that of joystick trials, and the modulation of non-BCI units might have diminished (Ganguly et al., 2011; Clancy et al., 2014). The present results most likely were obtained, therefore, while the monkeys still employed some degree of cognitive exploration that had not yet consolidated to automatic execution (Wander et al., 2013).

In controlling natural movements of the upper extremity, patterns of coactivation in M1 neurons are largely confined to an “intrinsic manifold” in the neural state-space (Sadtler et al., 2014; Gallego et al., 2018, 2020). Learning to control a BCI with M1 neurons progresses more quickly if the BCI decoder uses latent dimensions within this intrinsic manifold than if patterns outside the manifold are required (Sadtler et al., 2014). When such out-of-manifold patterns are required by the decoder, novel patterns of neural coactivation must be learned over many sessions (Oby et al., 2019). The time needed by the present monkeys to achieve our proficiency criteria for BCI performance, together with changes in the PDs and modulation depths of the BCI units, suggests that our BCI decoder most often constituted an out-of-manifold perturbation that required learning new patterns of unit coactivation. The newly learned patterns were not necessarily confined to the BCI units, and may have involved many non-BCI units in various cortical areas as well.

### Variability of changes observed

Although the present BCI units consistently showed changes in PD and modulation depth in all assignments, the concurrent changes observed in non-BCI units were comparatively variable across assignments, both within and between monkeys. Such variability might suggest that the activity of non-BCI units in some or all cortical areas was an epiphenomenon, irrelevant or even counter-productive to closed loop BCI performance. We consider this possibility to be unlikely, however. Although we cannot determine the exact causes of this variability, we suggest two potential factors that may have contributed.

First, as indicated by their success rates, response times, and cursor path lengths, the two monkeys approached the joystick and BCI tasks differently, with Monkey P performing better at the joystick task while Monkey Q achieved better performance at the BCI task. Our two subjects thus may have been at different positions on the spectrum from BCI learners to non-learners (Bridges et al., 2020). The behavioral differences between the two monkeys were accompanied by differences in neural activity. Non-BCI units in Monkey Q showed more consistent increases in the percent of units modulated, their modulation depths, and their effective connectivity with other units during BCI as compared with joystick trials than did non-BCI units in Monkey P. We speculate that the differences between the two monkeys in behavioral performance and in neural activity were interrelated, and were related as well to a difference in what appeared to be the monkey’s engagement in performing the BCI task.

Second, within a given animal, each different BCI-unit assignment likely required a different pattern of coactivation among the BCI units (Athalye et al., 2017; Oby et al., 2019). These different coactivation patterns among the BCI units may have been achieved with different patterns of activity in non-BCI units in the various cortical areas examined here, producing varying results from assignment to assignment within each subject. Although the BCI units were solely responsible for directly controlling the BCI output, a closed-loop BCI cannot be operated successfully without engaging the activity of at least some non-BCI units. The BCI units in our task must at least have received processed visual information about the location of the peripheral target and likely received visual feedback about the current location of the cursor. This information could only have come through non-BCI units, although not necessarily those that we recorded. In addition, internal decisions about when to initiate another trial probably influenced the BCI units, and their firing may also have provided efference copy to circuits comparing a forward model of the expected cursor trajectory with the actual incoming feedback. We speculate that, released from the need to control the motion of the cursor with movement of the native upper extremity, and with an expansive neural space available, the CNS found ways to provide these functions that varied to some extent from assignment to assignment.

### Effective connectivity of non-BCI units and BCI control

A previous study has shown that effective connectivity among the M1 BCI units controlling a reach-to-grasp robot changes progressively as non-human primates acquired proficient control, although the time course of these changes differed depending on whether the M1 BCI population was contralateral or ipsilateral to an upper extremity amputation (Balasubramanian et al., 2017). Here, we found that effective connectivity increased during BCI as compared with joystick control in one monkey but not the other. But in both monkeys, non-BCI units with effective connectivity to or from a BCI unit (Connect+ units) on average had higher *nMD*s during BCI trials than those without such connectivity (Connect– units). Our simulation (see Materials and Methods) indicates that this difference cannot be attributed simply to a higher likelihood of finding false positive connections for units with larger modulation depth. We therefore speculate that Connect+ non-BCI units are more likely than Connect– units to have played a relatively direct role in one or more of the ancillary functions required for closed-loop control—processing target location, inverse model, visual feedback, efference copy, forward model, etc. Although current concepts of corticocortical information flow during voluntary movement emphasize transmission of information from posterior parietal cortex to premotor cortex and then to M1 (Rizzolatti et al., 1998; Grafton, 2010), we found that effective connectivity between BCI and non-BCI units in most of the cortical areas we examined was largely bidirectional, both during joystick and during BCI trials. Our estimates of effective connectivity of course are based on statistical models and do not represent actual synaptic connectivity. But with the exception of dPPC, where we found no effective connectivity with BCI units during BCI trials, we found that similar fractions of non-BCI units in all cortical areas had effective connectivity to and effective connectivity from the BCI units in M1.

### Conclusions

The present study shows for the first time that changes in PD, modulation depth, and effective connectivity occur in units beyond the cortical area(s) that directly control a BCI and extend to many, although not all, cortical areas involved in the distributed cortical network for the sensorimotor control of voluntary movements. In theory, given that the performance of modern neuroprostheses falls short of natural control of a native limb (Hochberg et al., 2012; Rouse and Schieber, 2015; Wodlinger et al., 2015), harnessing the activity of units from multiple cortical areas in next generation BCIs might provide a more dexterous neuroprosthetic extremity. Further studies, ideally recording more units in each area, will be needed to extend the present findings to the more proficient performance achieved with BCI decoders optimized to incorporate the natural tuning of large numbers of neurons, and to determine if non-BCI units play similar or different functional roles in closed-loop control of the native limb versus a BCI.

## Synthesis

Reviewing Editor: Mark Laubach, American University Decisions are customarily a result of the Reviewing Editor and the peer reviewers coming together and discussing their recommendations until a consensus is reached. When revisions are invited, a fact-based synthesis statement explaining their decision and outlining what is needed to prepare a revision will be listed below. The following reviewer(s) agreed to reveal their identity: John Kalaska. Note: If this manuscript was transferred from JNeurosci and a decision was made to accept the manuscript without peer review, a brief statement to this effect will instead be what is listed below.

Your manuscript was reviewed by one of the two reviewers who saw it at JN. The reviewers comments are below. Please address this one concern in the Significance Statement, and also check the rest of the manuscript as suggested. Please communicate the precise changes that you make in your resubmission, and I will review it promptly. Thank you. -Mark

Reviewer #1:

The authors have responded positively and adequately to the comments from both reviewers. I have only one specific suggestion for a revision. The last sentence of the Significance Statement is:

“We suggest that multiple cortical areas may function similarly during voluntary closed-loop control of either the upper extremity or of a BCI.”

My concern is that this statement is potentially ambiguous and could be misinterpreted. I’m sure that they mean to state that each cortical area retains its own unique functional contribution to task performance whether the monkeys are using their arm or the BCI. Instead, the sentence could be misconstrued to mean that all the cortical regions are doing same thing, and it doesn’t matter what task the monkeys are performing. That is certainly not the message they want to convey.

The authors should also check if similar potentially-confusing statements are contained in the Abstract (the last sentence, for instance) and elsewhere in the main text.

## Author Response

We have copied the Reviewers’ comments here in plain font.

Our replies appear in Italics.

Manuscript Instructions

- The species studied is not mentioned in the abstract. Please make sure to update both the abstract in the article file and on the submission form.

We now have specified (line 42):

“In two subjects (Macaca mulatta)...”

---------------------------------------------

Synthesis of Reviews:

Computational Neuroscience Model Code Accessibility Comments for Author (Required):

N/A

Significance Statement Comments for Author (Required):

Please see comments from the reviewer on this section of the manuscript.

Comments on the Visual Abstract for Author (Required):

N/A

Synthesis Statement for Author (Required):

Your manuscript was reviewed by one of the two reviewers who saw it at JN. The reviewers comments are below. Please address this one concern in the Significance Statement, and also check the rest of the manuscript as suggested. Please communicate the precise changes that you make in your resubmission, and I will review it promptly. Thank you. -Mark

Reviewer #1:

The authors have responded positively and adequately to the comments from both reviewers. I have only one specific suggestion for a revision. The last sentence of the Significance Statement is:

“We suggest that multiple cortical areas may function similarly during voluntary closed-loop control of either the upper extremity or of a BCI.”

My concern is that this statement is potentially ambiguous and could be misinterpreted. I’m sure that they mean to state that each cortical area retains its own unique functional contribution to task performance whether the monkeys are using their arm or the BCI. Instead, the sentence could be misconstrued to mean that all the cortical regions are doing same thing, and it doesn’t matter what task the monkeys are performing. That is certainly not the message they want to convey.

Point taken. We have changed this sentence to (redlines 62-64, clean copy 61-62):

“We suggest that a given cortical area may function similarly during voluntary closed-loop control of either the upper extremity or a BCI.”

The authors should also check if similar potentially-confusing statements are contained in the Abstract (the last sentence, for instance) and elsewhere in the main text.

We have changed this sentence to (lines 51-52):

“During voluntary, closed-loop control, non-BCI units in a given cortical area may function similarly whether the effector is the native upper extremity or a BCI-controlled device.”

We also checked the remainder of the manuscript and found no other such ambiguities.

We also identified and corrected a few typographical errors, evident in the redlined version of the manuscript in lines 271, 432, 463, and 679.
